# An interactive plan and model evolution method for knowledge‐based pelvic VMAT planning

**DOI:** 10.1002/acm2.12403

**Published:** 2018-07-08

**Authors:** Meijiao Wang, Sha Li, Yuliang Huang, Haizhen Yue, Tian Li, Hao Wu, Song Gao, Yibao Zhang

**Affiliations:** ^1^ Key Laboratory of Carcinogenesis and Translational Research (Ministry of Education/Beijing) Department of Radiation Oncology Beijing Cancer Hospital & Institute Peking University Cancer Hospital & Institute Beijing China; ^2^ Department of Medical Physics Institute of Medical Humanities Peking University Beijing China; ^3^ Beijing City Key Lab for Medical Physics and Engineering School of Physics Institute of Heavy Ion Physics Peking University Beijing China

**Keywords:** knowledge‐based planning, model improvement, RapidPlan, rectal cancer

## Abstract

**Purpose:**

To test if a RapidPlan DVH estimation model and its training plans can be improved interactively through a closed‐loop evolution process.

**Methods and materials:**

Eighty‐one manual plans (P_0_) that were used to configure an initial rectal RapidPlan model (M_0_) were reoptimized using M_0_ (closed‐loop), yielding 81 P_1_ plans. The 75 improved P_1_ (P_1+_) and the remaining 6 P_0_ were used to configure model M_1_. The 81 training plans were reoptimized again using M_1_, producing 23 P_2_ plans that were superior to both their P_0_ and P_1_ forms (P_2+_). Hence, the knowledge base of model M_2_ composed of 6 P_0_, 52 P_1+_, and 23 P_2+_. Models were tested dosimetrically on 30 VMAT validation cases (P_v_) that were not used for training, yielding P_v_(M_0_), P_v_(M_1_), and P_v_(M_2_) respectively. The 30 P_v_ were also optimized by M_2_new_ as trained by the library of M_2_ and 30 P_v_(M_0_).

**Results:**

Based on comparable target dose coverage, the first closed‐loop reoptimization significantly (*P* < 0.01) reduced the 81 training plans’ mean dose to femoral head, urinary bladder, and small bowel by 2.65 Gy/15.63%, 2.06 Gy/8.11%, and 1.47 Gy/6.31% respectively, which were further reduced significantly (*P* < 0.01) in the second closed‐loop reoptimization by 0.04 Gy/0.28%, 0.18 Gy/0.77%, 0.22 Gy/1.01% respectively. However, open‐loop VMAT validations displayed more complex and intertwined plan quality changes: mean dose to urinary bladder and small bowel decreased monotonically using M_1_ (by 0.34 Gy/1.47%, 0.25 Gy/1.13%) and M_2_ (by 0.36 Gy/1.56%, 0.30 Gy/1.36%) than using M_0_. However, mean dose to femoral head increased by 0.81 Gy/6.64% (M_1_) and 0.91 Gy/7.46% (M_2_) than using M_0_. The overfitting problem was relieved by applying model M_2_new_.

**Conclusions:**

The RapidPlan model and its constituent plans can improve each other interactively through a closed‐loop evolution process. Incorporating new patients into the original training library can improve the RapidPlan model and the upcoming plans interactively.

## 1 INTRODUCTION

Knowledge‐based radiotherapy treatment planning is deemed to reduce the inter‐planner varieties of plan quality^1^, ^2^, ^3^, ^4^, ^5^, ^6^, ^7^, ^8^, ^9^, ^10^, ^11^, ^12^, ^13^ and expedite the planning process.^14^, ^15^, ^16^, ^17^The RapidPlan module in Eclipse treatment planning system of version 13.5 or later (Varian Medical Systems, Palo Alto, CA) has commercialized the knowledge‐based solution^18^, ^19^ and displayed good compatibility across patient orientations, treatment techniques, and systems.^20^, ^21^

Well‐trained RapidPlan models have outperformed conventional trial and error‐based manual planning by reducing excess organs‐at‐risk (OAR) dose with greater consistency.^17^, ^20^, ^22^, ^23^, ^24^, ^25^, ^26^, ^27^, ^28^, ^29^, ^30^ Should the model performance be highly dependent on the library volume^31^ and average quality of the training plans,^17^, ^32^ incorporating the model‐improved constituent training plans into the model (closed‐loop)^25^ may potentially evolve the model as a cycle of interactive improvement. There has been attempts to iteratively improve KDE (kernel density estimation)‐based DVH prediction model. However, compared with RapidPlan, the KDE algorithm did not consider division between in‐field and out‐of‐field regions, and the generated point objectives were tested on limited sample size based on Pinnacle (Philips Radiation Oncology Systems, Fitchburg, WI),^33^ whose optimization algorithm, progressive optimization algorithm (POA) is different from Eclipse's Photon Optimizer (PO). This study aims to evaluate the performance of the closed‐loop model evolution on rectal cancer patients in the environment of Eclipse RapidPlan V13.6 knowledge‐based treatment planning system.

## 2 MATERIALS AND METHODS

**Figure 1:**
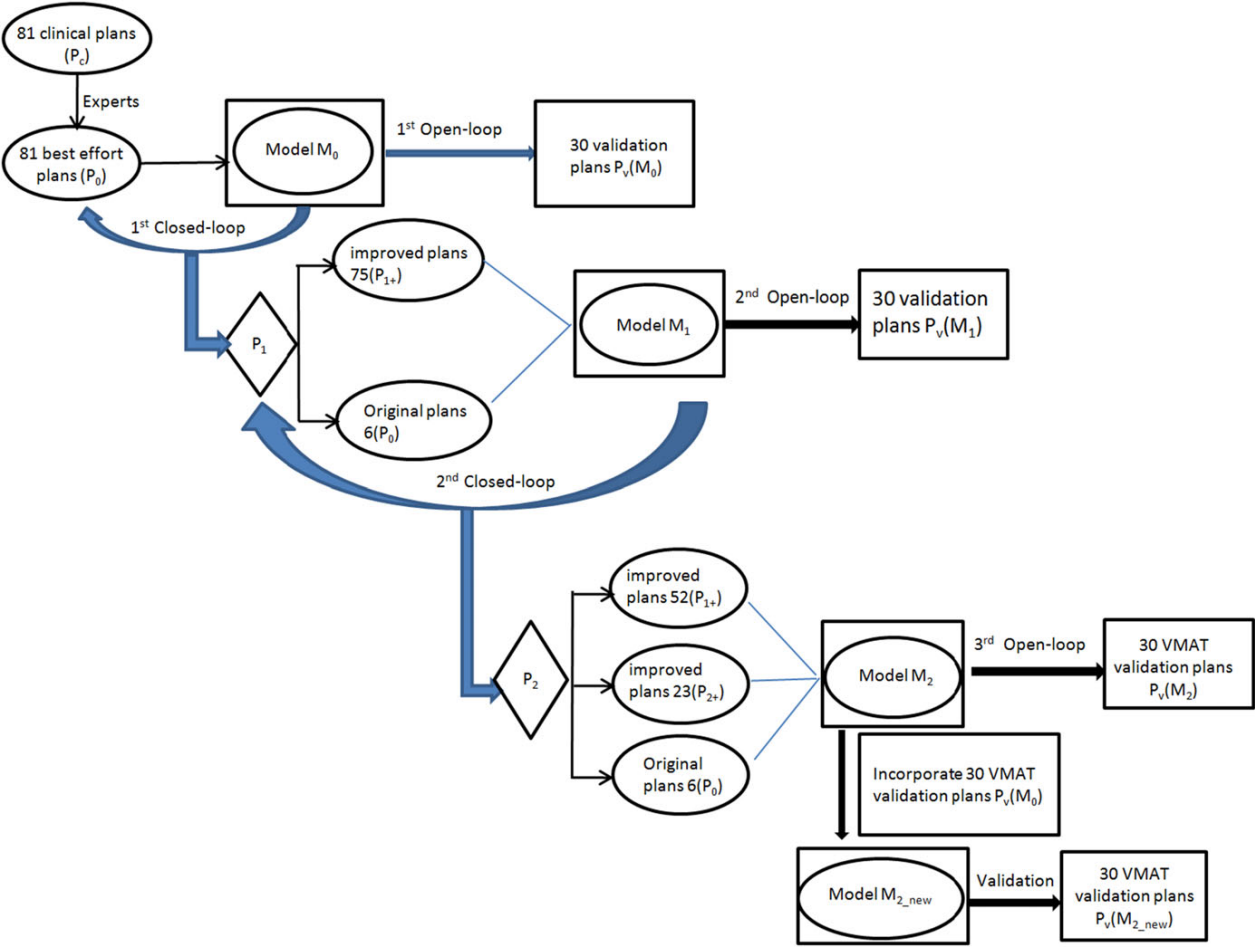
A schematic workflow and naming abbreviations of this work.

As a summary, Fig. 1 displays a schematic workflow explaining the evolution process and naming abbreviations.

### 2.A Initial model configuration

The planning and modeling details can be found in our previous publications.^17^, ^20^, ^34^ In summary, 81 clinical VMAT plans (P_c_) for preoperative rectal cancer patients were refined manually by experts (best‐effort manual plans, P_0_) to guarantee the initial plan quality and push a stricter evaluation criteria on the closed‐loop method. Plans were optimized to deliver 50.6 Gy and 41.8 Gy to 95% PTV_boost_ and 95% PTV respectively in 22 fractions.^35^ The extracted structure sets, prescriptions, and field geometries of P_0_ were regressed as the initial DVH estimation model (M_0_) and statistically verified using Varian Model Analytics tool.^36^ Model‐generated optimization objectives and priorities were assisted by additional manual constraints to make the model comply with our clinical protocols. The validations on 100+ patients have demonstrated that M_0_‐generated personalized objectives improved plan quality and consistency significantly compared to the clinical plans.^17^, ^20^

### 2.B Model evolution

As shown in Fig. 1, the 81 constituent P_0_ of M_0_ were reoptimized using M_0_ (closed‐loop), yielding training sets of first iteration (P_1_). To simplify the scoring of plan quality and avoid observer‐dependent evaluation preferences especially when the DVH lines have crossovers, three explicit endpoints: the mean dose to the femoral head, urinary bladder, and small bowel (*D*_mean_FH_, *D*_mean_UB_, and *D*_mean_SB_) were compared^33^. Plans with reduced *D*_mean_FH_, *D*_mean_UB_, and *D*_mean_SB_ were defined as improved plans (P_1+_). The first closed‐loop reoptimization using M_0_ produced 75 P_1+_, which composed M_1_ in addition to 6 P_0_ where the original plans were considered better. Second closed‐loop reoptimization using M_1_ derived 23 P_2+_ of better quality than both their P_0_ and P_1_ forms. The new model of each iteration was configured with best plans from all previous optimizations, hence M_2_ included 6 P_0_, 52 P_1+_, and 23 P_2+_. To be cost‐effective, iterations were terminated when no or clinically negligible improvement could be achieved anymore.

### 2.C Model assessment

To monitor the impact of modifying the knowledge base, open‐loop validation was performed on other 30 clinical VMAT plans (P_v_) that were not included in any model. All plans were renormalized to the target prescriptions before comparing the OAR exposure. Specifically, open‐loop validation on 30 P_v_ were reoptimized using M_0_, M_1,_ and M_2_, producing P_v_(M_0_), P_v_(M_1_), and P_v_(M_2_) respectively. The following metrics were evaluated: (a) homogeneity index ($\text{HI}=\left(D_{2\%}-D_{98\%}\right)/D_{50\%}$), where $D_{x\%}$ indicates the dose to *x*% of the volume; (b) conformity index ($\text{CI}=V_{100\%Rx}/V_{\mathrm{target}}$), where $V_{100\%Rx}$ and $V_{\mathrm{target}}$ indicate the volumes receiving at least 100% of the prescribed dose and the target volumes respectively; (c) the small bowel volumes receiving at least 35, 40, and 45 Gy (*V*_35Gy_SB_, *V*_40Gy_SB_, *V*_45Gy_SB_); (d) the femoral head and urinary bladder volumes receiving at least 40 and 45 Gy (*V*_40Gy_FH_, *V*_40Gy_UB_, *V*_45Gy_FH_, and *V*_45Gy_UB_); (e) The maximum and mean dose to the small bowel, femoral head, and urinary bladder (*D*_max_SB_, *D*_max_FH_, *D*_max_UB_; *D*_mean_SB_, *D*_mean_FH,_ and *D*_mean_UB_). (f) Using an in‐house MATLAB code, mean DVHs of 30 P_v_ and their reoptimized forms were calculated based on exported DVHs in tabular format, and were plotted for comparison using SigmaPlot software (v. 10.0, Systat, San Jose, CA).

To address the over‐fitting problem, the reoptimized 30 VMAT validation cases using M_0_ (P_v_(M_0_)) were added to the training library of M_2_, yielding model M_2_new_. The performance of M_2_new_ was tested on the 30 validation cases thereafter.

### 2.D Statistical methods

Using SPSS (v21.0, IBM Analytics, Armonk, NY), normality was tested using Shapiro–Wilk method. Normal and abnormal data were analyzed by paired samples *t*‐test and Wilcoxon signed‐rank test respectively (two‐tailed, significant level 0.05).

## 3 RESULTS

### 3.A Closed‐loop reoptimizations

After replacing the training library with 75 P_1+_ during the first closed‐loop refinement, the 81 plans used to configure model M_1_ were of comparable HI and CI (mean difference < 0.03) relative to the library of M_0_, but of consistently lower mean dose to all OARs. *D*_mean_FH_, *D*_mean_UB_, and *D*_mean_SB_ in M_1_ library were significantly reduced by 2.65 Gy (15.63%), 2.06 Gy (8.11%), and 1.47 Gy (6.31%) respectively (all *P* < 0.01), relative to M_0_ library.

After updating the library of model M_2_ with 23 P_2+_ that were superior to both P_0_ and P_1_ forms after the second closed‐loop reoptimization, the changes of HI and CI were negligible (mean difference < 0.03), yet the *D*_mean_FH_, *D*_mean_UB_, and *D*_mean_SB_ of 81 plans were further significantly reduced by 0.04 Gy (0.28%), 0.18 Gy (0.77%), 0.22 Gy (1.01%) on average respectively (all *P* < 0.01), relative to M_1_ library.

More details are shown in Tables 1 and 2.

**Table 1:** Dosimetric changes of 81 training plans after incorporating improved plans from the closed‐loop reoptimization: targets

	HI		CI	
	PTV_boost_	PTV	PTV_boost_	PTV
M_0_				
Mean ± SD	0.06 ± 0.01	0.26 ± 0.01	1.06 ± 0.07	1.02 ± 0.02
95% CI	0.06–0.06	0.26–0.27	1.05–1.08	1.02–1.03
M_1_				
Mean ± SD	0.05 ± 0.01	0.26 ± 0.01	1.09 ± 0.08	1.03 ± 0.02
95% CI	0.05–0.05	0.26–0.27	1.07–1.11	1.02–1.03
M_2_				
Mean ± SD	0.05 ± 0.01	0.26 ± 0.01	1.09 ± 0.08	1.03 ± 0.02
95% CI	0.05–0.05	0.26–0.27	1.07–1.10	1.02–1.03

HI, homogeneity index; CI, conformity index; PTV, planning target volume; M_x_, model after x round of closed‐loop refinement; SD, standard deviation; 95% CI, 95% confidence intervals.

**Table 2:** Dosimetric changes of 81 training plans after incorporating improved plans from the closed‐loop reoptimization: organs‐at‐risk

	M_0_	M_1_	M_2_
Femoral head			
*V*_40Gy_ (%)	0.03	0.01	0.01
*V*_45Gy_ (%)	0.00	0.00	0.00
*D*_max_ (Gy)	39.76	38.71	38.88
*D*_mean_ (Gy)	16.95	14.30	14.26
*P*	<0.01		<0.01
Urinary bladder			
*V*_40Gy_ (%)	16.22	14.46	14.43
*V*_45Gy_ (%)	3.28	4.20	4.15
*D*_max_ (Gy)	49.24	49.86	49.90
*D*_mean_ (Gy)	25.40	23.34	23.16
*P*	<0.01		<0.01
Small bowel			
*V*_35Gy_ (%)	3.89	4.75	4.55
*V*_40Gy_ (%)	0.14	0.34	0.34
*V*_45Gy_ (%)	0.00	0.00	0.00
*D*_max_ (Gy)	39.85	41.56	41.46
*D*_mean_ (Gy)	23.29	21.82	21.60
*P*	<0.01		<0.01

*V*_xGy_, volumes receiving at least x Gy; *D*_mean_, mean dose; *D*_max_, maximum dose; *P* values are for the comparisons of *D*_mean_; M_x_, model after x round of closed‐loop refinement.

### 3.B Validations

Based on 30 VMAT validation cases, knowledge‐based reoptimizations using various models yielded comparable target coverage (mean difference of HI and CI < 0.01), but the impact on the OARs were more complex and intertwined: relative to the results of using M_0_, monotonically increased magnitudes of mean dose reduction to two OARs were observed using the refined models M_1_ and M_2_, by 0.34 Gy (1.47%) and 0.36 Gy (1.56%) on average for urinary bladder, and by 0.25 Gy (1.13%) and 0.30 Gy (1.36%) on average for small bowel. However, M_1_ and M_2_ increased the mean dose to femoral head than M_0_, by 0.81 Gy (6.64%) and 0.91 Gy (7.46%) on average. More details are shown in Tables 3 and 4.

**Table 3:** The open‐loop validation results of various models on 30 additional patients: targets

	HI		CI	
	PTV_boost_	PTV	PTV_boost_	PTV
P_v_(M_0_)				
Mean ± SD	0.05 ± 0.01	0.27 ± 0.01	1.09 ± 0.05	1.05 ± 0.03
95% CI	0.05–0.05	0.26–0.27	1.07–1.11	1.03–1.06
P_v_(M_1_)				
Mean ± SD	0.05 ± 0.01	0.26 ± 0.01	1.09 ± 0.06	1.04 ± 0.03
95% CI	0.05–0.05	0.26–0.27	1.07–1.11	1.03–1.05
P_v_(M_2_)				
Mean ± SD	0.05 ± 0.01	0.27 ± 0.01	1.09 ± 0.06	1.04 ± 0.03
95% CI	0.05–0.05	0.26–0.27	1.07–1.11	1.03–1.05

HI, homogeneity index; CI, conformity index; PTV, planning target volume; P_v_(M_x_), validation plans reoptimized using model M_x_; M_x_, model after x round of closed‐loop refinement; SD, standard deviation; 95% CI, 95% confidence intervals.

**Table 4:** The open‐loop validation results of various models on 30 additional patients: organs‐at‐risk

	P_v_(M_0_)	P_v_(M_1_)	P_v_(M_2_)
Femoral head			
*V*_40Gy_ (%)	0.00	0.00	0.00
*V*_45Gy_ (%)	0.00	0.00	0.00
*D*_max_ (Gy)	37.42	38.29	38.07
*D*_mean_ (Gy)	12.20	13.01	13.11
*P*	<0.01		0.05
Urinary bladder			
*V*_40Gy_ (%)	12.94	12.87	12.85
*V*_45Gy_ (%)	2.84	2.83	2.78
*D*_max_ (Gy)	49.17	49.16	49.00
*D*_mean_ (Gy)	23.08	22.74	22.72
*P*	<0.01		0.66
Small bowel			
*V*_35Gy_ (%)	6.26	5.92	6.14
*V*_40Gy_ (%)	0.83	0.82	0.83
*V*_45Gy_ (%)	0.15	0.02	0.03
*D*_max_ (Gy)	42.51	42.51	42.75
*D*_mean_ (Gy)	22.10	21.85	21.80
*P*	0.39^a^		

P_v_(M_x_), validation plans reoptimized using model M_x_; M_x_, model after x round of closed‐loop refinement; *V*_xGy_, volumes receiving at least x Gy; *D*_max_, maximum dose; *D*_mean_, mean dose; *P* values are for the comparisons of *D*_mean_.

a Friedman test.

**Figure 2:**
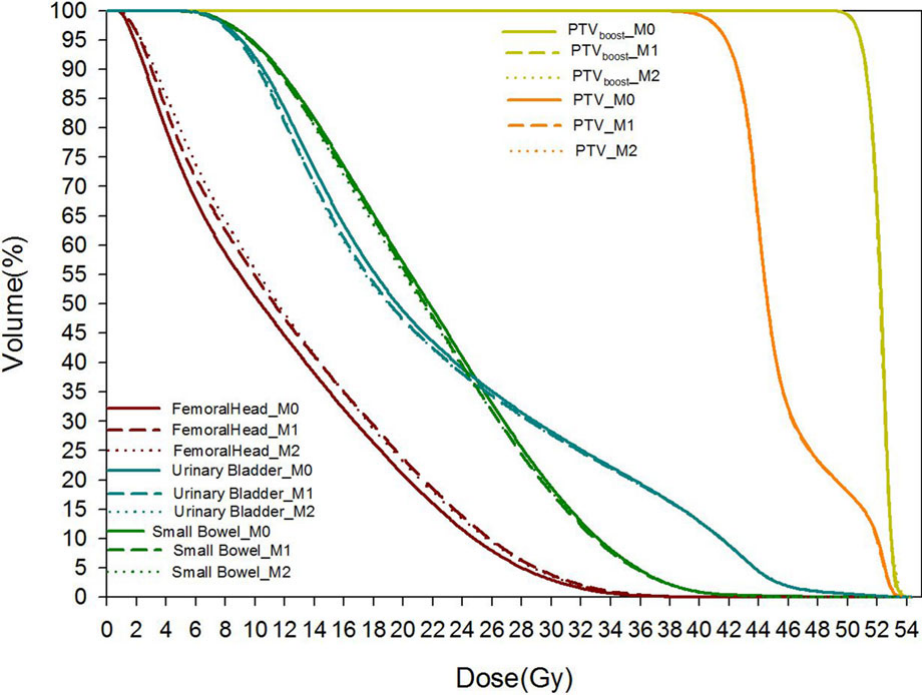
The mean DVH plots of 30 VMAT validation plans using various model M_0_, M_1_ and M_2_.

**Figure 3:**
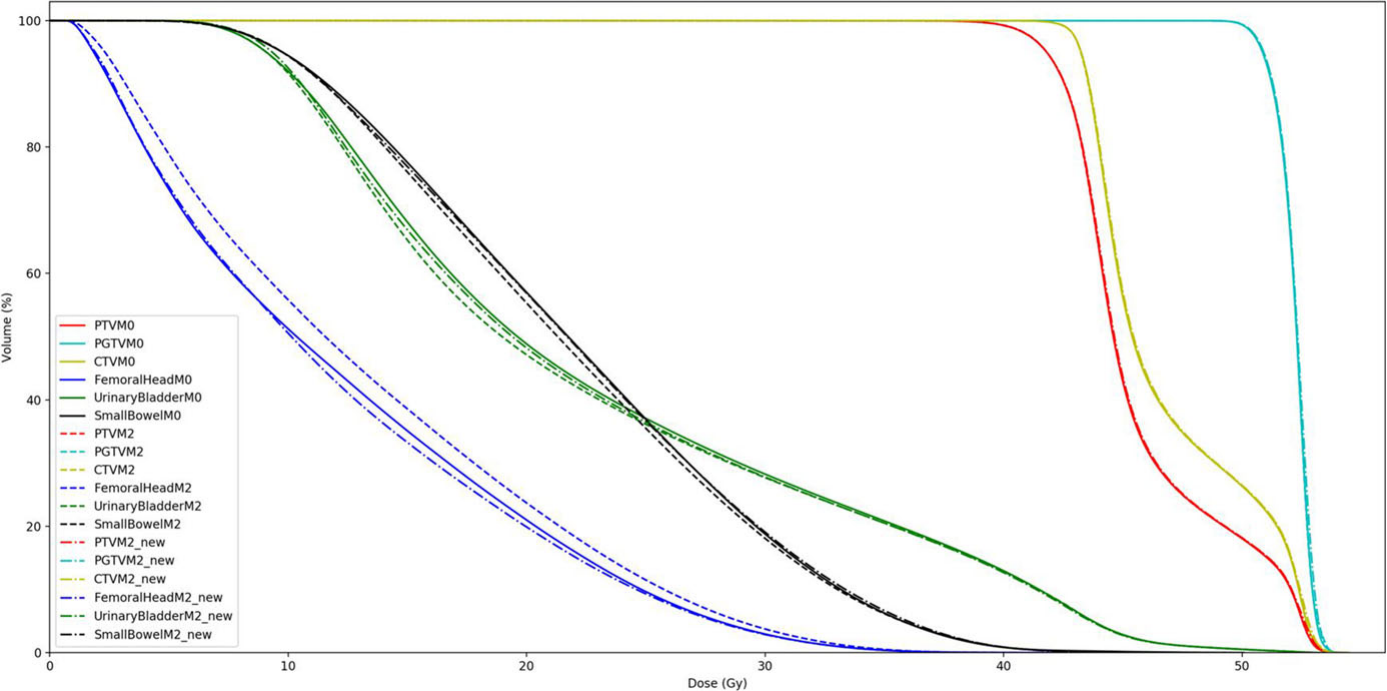
The mean DVH plots of 30 VMAT validation plans optimized using model M_0_, M_2_, and M_2_new_.

Figure 2 plots the mean DVHs of 30 VMAT P_v_ that were reoptimized using models M_0_, M_1_, and M_2_ respectively, as represented by the solid, dashed, and dotted lines. The largely overlapping DVHs of P_v_(M_1_) and P_v_(M_2_) were considered as indicators of terminating further iterations of closed‐loop model refinement. Figure 3 compares the mean DVHs of 30 P_v_ that were reoptimized using models M_0_, M_2_, and M_2_new_ respectively, as represented by the solid, dash, and dash‐dotted lines.

## 4 DISCUSSION

In the first closed‐loop refinement, most (75/81, 92.59%) of the best‐effort manual plans (P_0_) that were used to train the initial RapidPlan model (M_0_) can be further improved by closed‐loop knowledge‐based reoptimization using M_0_‐generated objectives. These results echoed the superiority of knowledge‐based solution over the conventional trial‐and‐error manual planning, in line with previous publications.^17^, ^20^, ^22^, ^23^, ^24^, ^25^, ^26^, ^27^ It suggested that knowledge‐ and geometry‐based dosimetric predictions can help avoid selecting suboptimal or conflict optimization constraints as manual limitations.

For 23 out of 81 training sets, excessive dose to all OARs were still deductable in the second closed‐loop reoptimization. However, the magnitudes of improvement were very marginal, which were treated as an indicator of iteration termination. It suggested exhausting potential of model evolution providing the anatomy and selected beam geometry.

To extend the geometric diversity and representativeness of the model, it is clinically desirable to enlarge the training set library by adding new appropriate cases. This work suggests that closed‐loop reoptimization of the new candidate is beneficial before the incorporation, to avoid introducing suboptimal plans into the knowledge base. Our solutions of updating the training library with improved plans may better preserve the anatomical variety, which distinguished our work from Li's work where poor plans were filtered.^37^ To avoid unacceptable geometric outliers, statistical verification assisted by Model Analytics^38^ can be helpful, where plans with Z‐scores > 3.5 were reviewed case‐by‐case. By adding the validation plans into the improved model library, the geometric representativeness were further improved.

The largely overlapping target DVHs in Fig. 2 echoed the comparable target numeric in Table 3, providing relatively fair basis for the comparison of the OAR dose in the open‐loop validation. However, incorporating the improved plans into the knowledge pool of M_1_ and M_2_ has made marginal and intertwined dosimetric changes in the open‐loop validation than using M_0_: two OARs were better spared using the models trained with better plans, at cost of excessive dose to one OAR though.

Three possible explanations could be: (a) RapidPlan generates line objectives under the lower bound of the estimation ranges (predicted DVH‐1 standard error), which already makes the optimization fairly challenging. The potential of dosimetric improvement based on the same patient anatomy and beam geometry may have been exhausted during the first round of knowledge‐based planning, making it less sensitive to the even lower estimation and objectives generated by the refined models. (b) A potential limitation of current RapidPlan algorithm may also be ascribed to: achievable DVH of each OAR was modeled and estimated independently, but OARs competed with each other hence optimal results can hardly be achieved simultaneously. Additional feedbacks such as patient‐specific adjustment of objective priorities might be beneficial to balance the complexity. Similar tradeoffs and overfitting problems were reported by Yuan, et al.^19^: although the training set was good, prediction errors between different OARs were still observed in some validation cases. The model generalization capability might be further improved by considering the OARs collectively, which is worthy of future studies. (c) Table 5 compares the anatomic statistics of the VMAT patients used for closed‐ and open‐loop iterations: although the ranges were largely overlapping, disparities were unavoidable because of the patient diversity. The similar patient anatomy and field geometry may partially explain the prevailed performance of closed‐loop than the open‐loop iterations. That is why it is clinically desirable to enlarge the model library continuously, and our closed‐loop evolution method can better preserve the training set volume.

**Table 5:** The anatomic statistics of the 81 model cases (closed‐loop) and 30 validation cases (open‐loop)

	81 model cases			30 validation cases		
	Min.	Max.	Mean	Min.	Max.	Mean
Total volume (cm^3^)						
PTV_boost_	53.31	618.09	179.54	65.99	474.06	170.69
PTV	844.12	1675.05	1207.99	776.29	1550.59	1090.8
Femoral head	95.82	334.91	199.88	108.43	346.00	241.89
Urinary bladder	55.19	744.34	283.19	63.88	693.7	250.41
Small bowel	60.27	1152.52	457.90	120.69	1025.27	517.89
Overlap with targets (cm^3^)						
Femoral head	0	0.05	0	0	0	0
Urinary bladder	0.29	161.44	38.18	0.11	107.48	32.71
Small bowel	0	0	0	0	0	0
Overlap with targets (%)						
Femoral head	0	0.02	0	0	0	0
Urinary bladder	0.21	39.01	13.70	0.17	51.06	12.40
Small bowel	0	0	0	0	0	0

Min., minimum; Max., maximum.

The overfitting problem of Fig. 2 can be relieved by Model M_2_new_. As shown in Fig. 3, the reoptimized P_v_ using M_2_new_ spared femoral head better at comparable if not lower dose to the other two OARs than the results of M_0_. This might be ascribed to the improved geometric similarities of M_2_new_ with P_v_, which can be applied clinically to further improve knowledge‐based planning. Considering the effects of overfitting reduction, similar methodology of incorporating optimized plans into model training library and then reoptimizing the plan is recommended in future clinical practice.

As a preliminary feasibility study, this work is limited by the single treatment site and single treatment technique. Further work is anticipated in the future to validate the extensibility of the method in a clinical environment. In addition, the extra workload of refining the training library with new patients can be partially conducted by a scripting program, which will be developed in the future.

## 5 CONCLUSIONS

Based on a rectal RapidPlan model, this study demonstrated that the constituent plans used to develop the DVH estimation model can be interactively improved by the model in a closed‐loop reoptimization. Incorporating new patients into the original training library can improve the RapidPlan model and the upcoming plans interactively.

## CONFLICT OF INTEREST

This work was partially supported by Varian Research Collaboration Grant. Dr. Yibao Zhang and Mr. Hao Wu received speaker's honorarium from Varian Medical Systems.
